# Prostaglandin D_2_ metabolite in urine is an index of food allergy

**DOI:** 10.1038/s41598-017-17798-w

**Published:** 2017-12-15

**Authors:** Shingo Maeda, Tatsuro Nakamura, Hiroaki Harada, Yuri Tachibana, Kosuke Aritake, Tatsuo Shimosawa, Yutaka Yatomi, Takahisa Murata

**Affiliations:** 1 Department of Animal Radiology, Graduate School of Agricultural and Life Sciences, The University of Tokyo, Tokyo, Japan; 2 Department of Allergy and Rheumatology, Graduate School of Medicine, The University of Tokyo, Tokyo, Japan; 3 International Institute for Integrative Sleep Medicine, University of Tsukuba, Ibaraki, Japan; 4 Department of Clinical Laboratory Medicine, Graduate School of Medicine, The University of Tokyo, Tokyo, Japan

## Abstract

Food allergy is immediate hypersensitive reactions to ingested foods. Since early diagnosis is effective for disease control, development of an objective diagnostic index is required. Using mediator-lipidomics, we found that levels of the urinary prostaglandin D_2_ (PGD_2_) metabolite, tetranor-PGDM, reflected the severity of the allergic symptoms and intestinal mast cell hyperplasia in mice. Repeated oral challenges with ovalbumin promoted allergic symptoms in sensitized mice. Particularly, the allergic mice presented with increased numbers of intestinal mast cells, which strongly expressed hematopoietic PGD synthase (H-PGDS). The levels of urinary tetranor-PGDM increased as the disease progressed. Treatment with a mast cell inactivator or an anti-inflammatory steroid attenuated these symptoms and decreased the tetranor-PGDM urinary levels. The levels of urinary tetranor-PGDM did not correlate with the disease severity in murine models of colitis, asthma, or allergic dermatitis. Furthermore, we have shown that urinary levels of tetranor-PGDM were significantly higher in patients with food allergy than those in healthy volunteers and patients with other types of allergic diseases such as asthma, allergic rhinitis, and atopic dermatitis. These findings suggest that urinary tetranor-PGDM is a useful diagnostic index of food allergy in both mice and humans.

## Introduction

Allergy to foods is a major health concern; currently, there is a global increase in patients with food allergy. In the United States, about 1–3% of adults and 3–8% of young children suffer from food allergy^1–3^. Most patients with food allergy are divided into antigen-specific IgE dependent type I hyper-sensitivity. Intake of food allergens activates mast cells that bind to allergen-specific IgE on their surface and induces the clinical manifestations of food allergy, including vomiting, diarrhea, urticaria, and occasionally fatal systemic anaphylaxis.

Early diagnosis is important for the effective control of food allergy; e.g. allergen exclusion and immunotherapy. Recent studies showed that the early consumption of food antigen significantly decreased the development of allergic disease in high-risk infants^4,5^. Diagnosis of food allergy is usually based on a history of food consumption, which is further supported by detection of serum food-specific IgE and/or positive skin prick tests. Although these IgE examinations are indicative of food allergen sensitization, in some cases, they fail to reflect clinical manifestations^6^. It can also be a physical and psychological burden to collect blood and perform skin prick tests, especially in young children. Oral food challenge (OFC) is the only definitive diagnostic method for food allergy^7^. However, this procedure is not commonly performed because anaphylactic reactions could be elicited during the challenge. Thus, developing more convenient and better diagnostic strategies is necessary.

Mediator lipidomics using liquid chromatography-tandem mass spectrometry (LC-MS/MS) has been recently developed and enables us to analyze bio-active lipid mediators more sensitively and comprehensively. It is very useful to investigate diseases and discover disease indices^8,9^. Using this technology, we hereby attempted to explore urinary diagnostic index for food allergy, as it can be safely and easily monitored even in pediatric patients.

In this study, we discovered that the urinary content of tetranor-prostaglandin D metabolite (tetranor-PGDM), a metabolite of mast cell-derived PGD_2_^10,11^, positively correlated with the severity of symptoms in a murine model of food allergy. We also found high levels of tetranor-PGDM in patients with food allergy but not in those with other allergic diseases or healthy volunteers. These results suggest the efficacy of urinary tetranor-PGDM as a potential index for diagnosis and therapeutic monitoring of food allergy.

## Results

### Urinary tetranor-PGDM levels reflect the severity of allergic symptoms and intestinal mast cell number in food allergy mouse models

**Figure 1:**
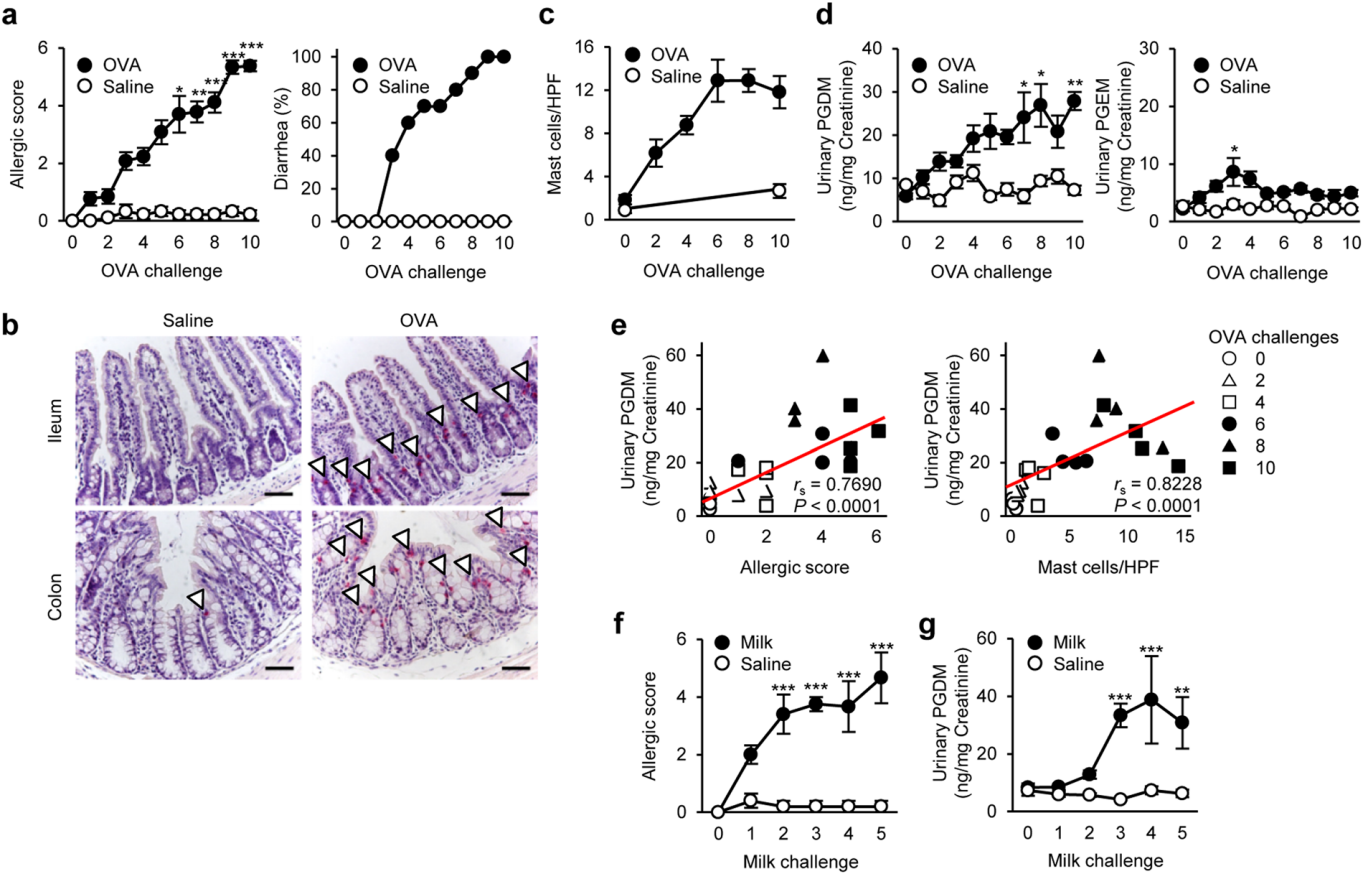
Urinary tetranor-PGDM is an index of food allergy. (**a**) Systemic allergic score (left) and diarrhea occurrence (right) in oral OVA- or saline-challenged BALB/c WT mice (*n* = 9–13). Kruskal–Wallis test with the Steel-Dwass test was used. ^*^ *P* < 0.05, ^**^ *P* < 0.01, and ^***^ *P* < 0.001 vs. saline-challenged mice. (**b**) Chloroacetate esterase (CAE)-stained ileum and colon sections following the tenth OVA-challenge. Arrowheads indicate mast cells. Scale bar: 50 μm. (**c**) Mast cell number per high-power field (HPF) in the colon of OVA- or saline-challenged mice (*n* = 4–8). (**d**) Tetranor-PGDM (left) and tetranor-PGEM (right) in urine of OVA- or saline-challenged mice (*n* = 5–10). One-way ANOVA with Tukey’s test was used. ^*^ *P* < 0.05 and ^**^ *P* < 0.01 vs. saline-challenged mice. (**e**) Correlation between urinary tetranor-PGDM level and allergic score (left) or intestinal mast cell number (right) in OVA-challenged mice (*n* = 4). Spearman’s rank correlation coefficient was used. (**f**) Systemic allergic score in oral milk- or saline-challenged mice (*n* = 5 each). Kruskal-Wallis test with the Steel-Dwass test was used. ^***^ *P* < 0.001 vs. saline-challenged mice. (**g**) Urinary tetranor-PGDM in oral milk- or saline-challenged mice (*n* = 5 each). One-way ANOVA with Tukey’s test was used. ^**^ *P* < 0.01 and ^***^ *P* < 0.001 vs. saline-challenged mice.

To explore urinary index for food allergy, we initially established an oral allergen-induced food allergy mouse model^12^. Repeated oral challenges of ovalbumin (OVA) gradually elicited systemic allergic responses including scratching, immobility, swelling and diarrhea in sensitized mice (Fig. 1a). Histological studies via chloroacetate esterase (CAE) staining, which can detect intestinal mast cells, showed that mast cells infiltrated into intraepithelium in both small and large intestines upon OVA-challenge (Fig. 1b and Supplemental Fig. S1a). The number of intestinal mast cells gradually increased in conjunction with disease progression (Fig. 1c).

Using urine from the food allergy model mice, we performed comprehensive analysis of lipid metabolites by LC-MS/MS and calculated the relative ratios of lipid metabolites in the OVA-challenged mice versus unchallenged mice (Supplemental Fig. S2). Among 25 metabolites identified in the urine, we found that tetranor-PGDM, 20-hydroxy PGE_2_, 8-iso PGF_3α_, and oleoylethanolamide (OEA) showed gradual increases in mice with food allergy (Supplemental Fig. S2). Urinary tetranor-PGEM, tetranor-PGFM, and lipoxin A_5_ showed transient increases that peaked at the third OVA-challenge (Supplemental Fig. S2). Since tetranor-PGDM, an arachidonic acid-derived PGD_2_ metabolite, showed the greatest increase during the OVA-challenges (Supplemental Fig. S2), we assessed the utility of this metabolite as a urinary index for food allergy in subsequent experiments. Metabolic pathway of tetranor-PGDM is shown in Supplemental Fig. S3. The urinary levels of tetranor-PGDM significantly increased in accordance with the disease progression (Fig. 1d). Its concentration positively correlated with the allergic score and also with intestinal mast cell number (Fig. 1e). In contrast, the urinary concentration of a metabolite of a major inflammatory mediator PGE_2_, tetranor-PGEM, transiently and slightly increased after the early challenges (Fig. 1d).

Repeated milk-challenges also caused systemic allergic responses accompanied by intestinal mast cell hyperplasia (Fig. 1f and Supplemental Fig. S1b). A gradual increase in the urinary level of tetranor-PGDM was observed in this allergic model as well (Fig. 1g). These results suggest that urinary levels of tetranor-PGDM reflect the disease severity and extent of mast cell hyperplasia in a murine model of food allergy, regardless of antigen-type.

### Urinary tetranor-PGDM is derived from mast cell-producing PGD_2_

**Figure 2:**
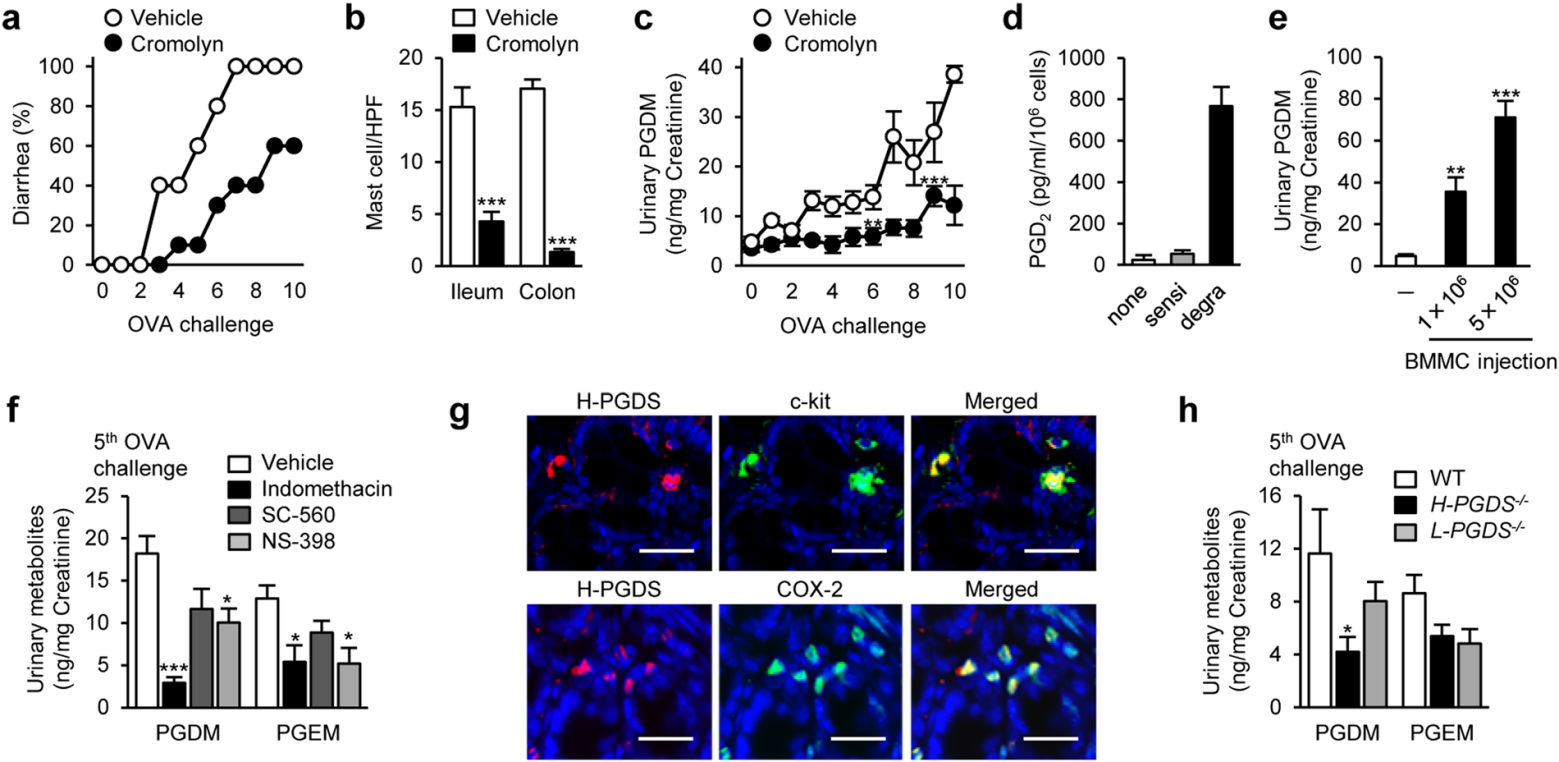
Cellular and enzymatic source of tetranor-PGDM in food allergy. (**a**) Diarrhea occurrence in OVA-induced food allergic mice treated with cromolyn (15 mg/kg) or vehicle (*n* = 5 each). (**b**) Mean number of intestinal mast cells in OVA-induced food allergic mice treated with cromolyn (15 mg/kg) or vehicle (*n* = 4 each). Student’s *t* test was used. ^***^ *P* < 0.001 vs. vehicle-treated mice. (**c**) Urinary tetranor-PGDM level in OVA-induced food allergic mice treated with cromolyn (15 mg/kg) or vehicle (*n* = 5 each). One-way ANOVA with Tukey’s test was used. ^**^ *P* < 0.01 and ^***^ *P* < 0.001 vs. vehicle-treated mice. (**d**) PGD_2_ production in naive, sensitized, and degranulated BMMCs *in vitro* (*n* = 4 each). (**e**) Urinary tetranor-PGDM in naïve or BMMC transferred *Kit* ^*W-sh/W-sh*^ mice (*n* = 5 each). One-way ANOVA with Dunnett’s test was used. ^**^ *P* < 0.01 and ^***^ *P* < 0.001 vs. naïve mice. (**f**) Urinary tetranor-PGDM and tetranor-PGEM levels in fifth OVA-challenged mice with either vehicle, indomethacin (5 mg/kg), SC-560 (10 mg/kg), or NS-398 (10 mg/kg) (*n* = 5 each). One-way ANOVA with Dunnett’s test was used. ^*^ *P* < 0.05 and ^***^ *P* < 0.001 vs. vehicle-treated mice. (**g**) Immunostained colon sections with H-PGDS (red) and c-kit (green, upper) or COX-2 (green, lower) following the tenth OVA-challenge. Scale bar: 25 μm. (**h**) Urinary tetranor-PGDM level in fifth OVA-challenged WT, *H-PGDS* ^−/−^, and *L-PGDS* ^−/−^ mice (*n* = 5–8). One-way ANOVA with Dunnett’s test was used. ^*^ *P* < 0.05 vs. WT mice.

Mast cells are known to release a large scale of PGD_2_^13^. Administration of a mast cell stabilizer, cromolyn (15 mg/kg, daily), attenuated the allergic diarrhea, reduced intestinal mast cell number, and decreased the concentration of urinary tetranor-PGDM in the OVA-challenged food allergic mice (Fig. 2a–c). *In vitro*, bone marrow-derived murine mast cells (BMMCs) degranulated by antigen-stimulation released PGD_2_ (Fig. 2d). *Kit* ^*W-sh/W-sh*^ mice, which lack mature mast cells due to an inversion mutation in the *Kit* gene promoter^14^, exhibited very low levels of urinary tetranor-PGDM (Fig. 2e). Single injection of BMMCs (1–5 × 10^6^ cells) into *Kit* ^*W-sh/W-sh*^ mice significantly increased urinary tetranor-PGDM level in a cell number-dependent manner (Fig. 2e). However, its level is relatively high, single injection of many mast cells may unphysiologically degranulate them.

PGD_2_ is produced by cyclooxygenases (COX) and PGD synthase (PGDS)^15^. There are two types of COX–constitutive type (COX-1) and inducible type (COX-2) and two types of PGDS–hematopoietic-type (H-PGDS) and lipocalin-type (L-PGDS). Administration of a non-selective COX inhibitor, indomethacin (5 mg/kg, daily), or a COX-2 selective inhibitor, NS-398 (10 mg/kg, daily), significantly decreased the urinary levels of both tetranor-PGDM and tetranor-PGEM in the food allergic mice (Fig. 2f). However, a COX-1 selective inhibitor, SC-560 (10 mg/kg, daily), slightly, but not significantly, decreased these urinary metabolites (Fig. 2f). Immunofluorescence analysis showed that c-kit-positive intestinal mast cells strongly expressed H-PGDS (Fig. 2g, upper). Most H-PGDS-positive cells expressed COX-2 (Fig. 2g, lower). Consistently, H-PGDS deficiency, but not L-PGDS deficiency, significantly decreased the urinary level of tetranor-PGDM in the food allergic mice (Fig. 2h).

Taken together, these results suggest that the production and excretion of urinary tetranor-PGDM in food allergy is attributable to intestinal mast cell-derived PGD_2_ through the activity of COX-2 and H-PGDS.

### Urinary tetranor-PGDM levels in other disease models

**Figure 3:**
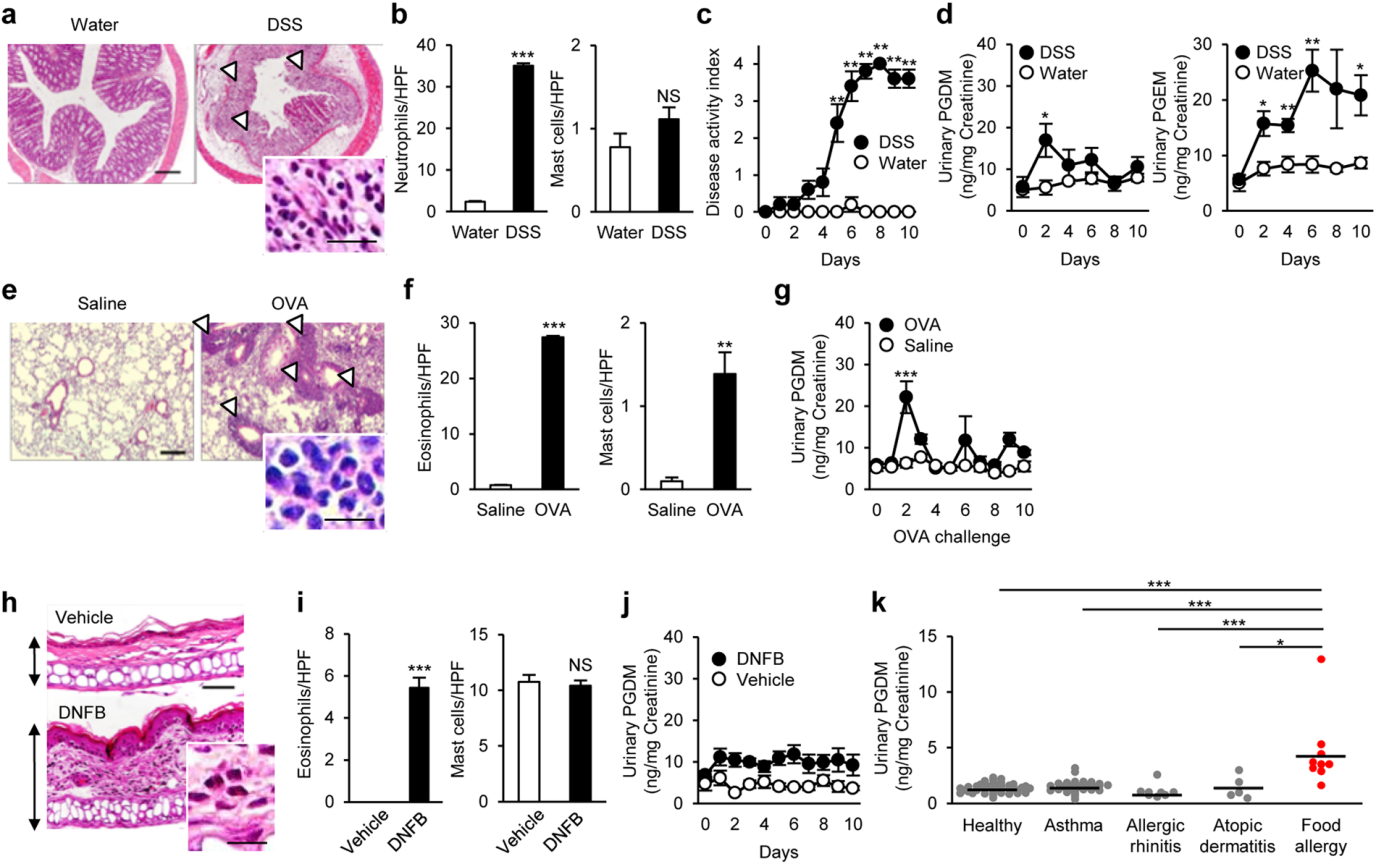
Urinary tetranor-PGDM in other disease models and human patients. (**a**) H&E-stained colon sections in DSS-induced colitis. Arrowheads indicate mucosal damages. Scale bar: 200 μm. Inset: Neutrophil infiltration. Scale bar: 25 μm. (**b**) Mean number of colonic neutrophils (left) and mast cells (right) in DSS- or water-treated mice (*n* = 5 each). Student’s *t* test was used. ^***^ *P* < 0.001 vs. water-treated mice. (**c**) Disease activity index in DSS- or water-treated mice (*n* = 5 each). Kruskal-Wallis test with the Steel-Dwass test was used. ^**^ *P* < 0.01 vs. water-treated mice. (**d**) Urinary tetranor-PGDM (left) and tetranor-PGEM (right) in DSS- or water-treated mice (*n* = 5 each). One-way ANOVA with Tukey’s test was used. ^*^ *P* < 0.05 and ^**^ *P* < 0.01 vs. water-treated mice. (**e**) H&E-stained lung sections in the allergic airway inflammation. Arrowheads indicate leukocyte accumulation. Scale bar: 200 μm. Inset: Eosinophil infiltration. Scale bar: 25 μm. (**f**) Mean number of lung eosinophils (left) and mast cells (right) in intranasal OVA- or saline-challenged mice (*n* = 5–7). Student’s *t* test was used. ^**^ *P* < 0.01 and ^***^ *P* < 0.001 vs. saline-challenged mice. (**g**) Urinary tetranor-PGDM in intranasal OVA- or saline-challenged mice (*n* = 5–7). One-way ANOVA with Tukey’s test was used. ^***^ *P* < 0.001 vs. saline-challenged mice. (**h**) H&E-stained ear sections in the allergic dermatitis. Arrows indicate ear thickening. Scale bar: 50 μm. Inset: Eosinophil infiltration. Scale bar: 10 μm. (**i**) Mean number of eosinophils (left) and mast cells (right) in the ears of DNFB- or vehicle-treated mice (*n* = 5 each). Student’s *t* test was used. ^***^ *P* < 0.001 vs. vehicle-treated mice. (**j**) Urinary tetranor-PGDM in DNFB- or vehicle-treated mice (*n* = 5 each). One-way ANOVA was used. (**k**) Urinary tetranor-PGDM in healthy human volunteers (*n* = 39) and patients with asthma (*n* = 37), allergic rhinitis (*n* = 8), atopic dermatitis (*n* = 5), and food allergy (*n* = 9). The mean of the levels of tetranor-PGDM in each group is denoted by horizontal lines. Kruskal-Wallis test with the Steel-Dwass test was used. ^*^ *P* < 0.05 and ^***^ *P* < 0.001 vs. patients with food allergy.

We next examined the excretion of urinary tetranor-PGDM in non-allergic intestinal inflammation model mice. Administration of dextran sodium sulfate (DSS) caused severe intestinal inflammation with infiltration of neutrophils into the damaged mucosa (Fig. 3a,b). However, DSS administration did not influence the number of intestinal mast cells (Fig. 3b). Disease activity index based on fecal conditions was gradually increased in the DSS-treated mice (Fig. 3c). In these mice, urinary tetranor-PGDM showed slight increase just after the beginning of administration, while the inflammatory indicator, tetranor-PGEM was gradually and remarkably higher (Fig. 3d).

We further examined the urinary levels of tetranor-PGDM in other allergic models, allergic airway and skin inflammation. Repeated intranasal OVA-challenges induced allergic airway inflammation with accumulation of leukocytes, mainly eosinophils characterized by acidophilic cytoplasm and segmented nucleus, around the bronchus and blood vessel in lungs of sensitized mice (Fig. 3e). Mast cells were also significantly higher in the lungs, while the infiltration of eosinophils was more severe than that of mast cells (Fig. 3f). In this model, transient increase of tetranor-PGDM was observed only during the early OVA-challenges (Fig. 3g). Application of 2,4-dinitrofluorobenzene (DNFB) caused ear swelling accompanied with infiltration of eosinophils but not mast cells in DNP-IgE sensitized mouse pinna (Fig. 3h,i). In this allergic dermatitis model, the urinary level of tetranor-PGDM did not change during the disease progression (Fig. 3j). Thus, the patterns of tetranor-PGDM in mice with colitis, allergic airway inflammation, or allergic dermatitis were distinct from those in the food allergic mice.

### Urinary tetranor-PGDM and tetranor-PGEM levels in human patients with food allergy

Finally, we examined urinary levels of tetranor-PGDM and tetranor-PGEM in allergic patients (Fig. 3k and Supplemental Fig. S4). These patients did not show prominent symptoms at the time of urine collection. As in the murine models, urinary levels of tetranor-PGDM in patients with food allergy were significantly higher than those in healthy volunteers and other allergic patients, including asthma, allergic rhinitis and atopic dermatitis (Fig. 3k). In contrast, there was no significant difference in the levels of urinary tetranor-PGEM among healthy and allergic patients (Supplemental Fig. S4).

## Discussion

In this study, we identified urinary tetranor-PGDM as a food allergy-specific, disease severity-associated, and treatment-responsive index. Urinary tetranor-PGDM will be a convenient tool to monitor the severity of allergic symptoms. In addition, its sample can be collected non-invasively.

Allergen-specific IgE testing is widely used to identify causative food allergens and is useful for the diagnosis of food allergy^16,17^. The amount of IgE can indicate the probability of the incidence of symptoms. However, IgE levels sometimes do not reflect the development of clinical allergy. In a survey conducted in the United States, 16.8% of the participants were positive for serum IgE against peanut, cow milk, egg white, or shrimp, while only 2.5% of them had clinical symptoms^18^. Therefore, there is a need to develop more sensitive and specific markers for food allergy. We have found that the level of urinary tetranor-PGDM correlates with the disease severity and mast cell number in murine models of food allergy. Thus, urinary tetranor-PGDM may be a useful index for specific and sensitive diagnosis of food allergy.

Two major subsets of mast cells have been described: connective tissue-type mast cells (CTMCs) and mucosal-type mast cells (MMCs)^19^. CTMCs constitutively reside in the skin and intestinal submucosa, whereas MMCs are inducible upon stimulation and reside in the mucosal region. Both types of mast cells strongly express H-PGDS and produce considerably higher amounts of PGD_2_ than other types of immune cells, such as platelets, macrophages, dendritic cells, and certain T lymphocytes^20,21^. During the progression of food allergy, a number of mast cells, especially intraepithelial MMCs, extensively infiltrate into both small and large intestinal mucosa, leading to the remarkable increase of tetranor-PGDM content in the urine. In contrast, the slight increase of urinary tetranor-PGDM was observed in non-allergic intestinal inflammation and other allergic models. These differences may be due to the differences in type and number of contributing cells among various types of diseases. Neutrophils and eosinophils but not mast cells were infiltrated into the inflammatory lesions in mice with colitis and allergic dermatitis, respectively. Although the number of mast cells was significantly higher in the lungs of mice with allergic airway inflammation, the number of mast cells was far less than that in the intestines of food allergic mice. Food allergy is likely to be highly dependent on mast cells, which constitute a large area of intestine compared with the other types of diseases^12^.

This study showed that PGD_2_ production and thereby excretion of urinary tetranor-PGDM at the onset of allergic reactions are caused by the activation of COX-2 and H-PGDS in food allergy model mice. Since the levels of tetranor-PGDM are influenced by COX inhibitions, we need attention to interpret the levels in patients using nonsteroidal anti-inflammatory drugs. Further investigations will be necessary to examine the effects of medication on the levels of urinary tetranor-PGDM in humans.

PGE_2_ is the most prominent pro-inflammatory mediator that is involved in various types of inflammatory diseases^22^. Previous study has shown that urinary level of tetranor-PGEM is significantly increased upon viral infectious fever^23^. We also found the elevation of urinary tetranor-PGEM in mice with colitis, whereas the food allergic mice showed slight and transient increase in the levels. The induction of other allergic diseases, airway inflammation and dermatitis, did not influence the levels of tetranor-PGEM in this study (data not shown). These results indicated that food allergic inflammation can be distinguished from other types of inflammatory diseases based on urinary tetranor-PGEM levels. Further studies are necessary to confirm the utility of the simultaneous analysis of urinary tetranor-PGDM and tetranor-PGEM for accurate diagnosis of food allergy.

In clinical practice, OFC and immunotherapy are performed for definitive diagnosis of food allergy and complete oral tolerance, respectively^5,7,24–26^. Results of OFC and immunotherapy are evaluated based on subjective allergic symptoms after challenge with the suspected allergen, which may be interpreted erroneously by observers. Urinary tetranor-PGDM could be an objective indicator to evaluate the clinical reactivity observed in OFC and immunotherapy.

In summary, we have discovered that urinary tetranor-PGDM monitors intestinal mast cell accumulation and thereby reflects the severity of allergic symptoms in a murine model of food allergy. Human patients with food allergy also exhibited high levels of urinary tetranor-PGDM. Although the causative food allergen cannot be identified by measuring this disease index, an accurate diagnosis can be obtained by concomitant measurement of urinary tetranor-PGDM and allergen-specific IgE. Our findings provide new insights into mast cell biology and the diagnostic potential of urinary tetranor-PGDM in food allergies.

## Methods

### Mice

BALB/c wild type (WT) mice were obtained from CLEA Japan (Tokyo, Japan). *Kit* ^*W-sh/W-sh*^ mice were provided by the RIKEN BioResource Center (Tsukuba, Japan). *H-PGDS* ^−/−^ and *L-PGDS* ^−/−^ mice were generated and bred as previously described^27,28^. Mice were maintained at a 12-hour dark cycle at 22 °C and had free access to food and water. All mouse experiments were approved by the Institutional Animal Care and Use Committee of the University of Tokyo (No. 1034-2833 and P16-201). All experimental methods were performed in accordance with the approved guidelines.

### Patients

Allergic patients who regularly attend the University of Tokyo Hospital were enrolled. The patients were clinically diagnosed with one of the following allergic diseases: asthma, allergic rhinitis, and atopic dermatitis. Patients with food allergy were diagnosed based on either an oral food challenge or a history of hypersensitive reactions to particular foods via the presence of a specific IgE response as determined by skin prick testing or a commercial immunoassay. Patients that had been treated with corticosteroids and non-steroidal anti-inflammatory drugs in the 2 weeks before the urine collection were excluded. All subjects provided informed consent, and the study protocol was approved by the Ethics Committee of the University of Tokyo (No. 10586-1). All experiments were performed in accordance with the approved guidelines.

### Food allergy models

BALB/c mice were sensitized twice on day 1 and 14 by intraperitoneal injection with 50 μg of OVA (Sigma-Aldrich, St. Louis, MO) and 1 mg of aluminum potassium sulfate (Sigma-Aldrich) in a total volume of 100 μL saline. Sensitized mice were challenged orally with 10 mg of OVA in 100 μL saline or saline only every other day from day 28. Allergic symptoms were assessed by monitoring mice for 1 h following oral challenge and scored based on four criteria: diarrhea, scratching behavior, loss of mobility, and puffiness, as previously described with a slight modification^29^. Diarrhea was scored as follows: normal stool = 0, soft = 1, and diarrhea = 2. Scratching was graded based on the average number of scratching episodes during each 15 min interval as follows: 0–3 episodes = 0, 4–5 episodes = 1, and >6 episodes = 2. Loss of mobility was graded in duration of immobility as follows: <10 min = 0, <30 min = 1, and >30 min = 2. Puffiness, including bristled fur, edema around the nose and eyes, or laborious breathing, was scored as follows: none = 0 and puffiness = 2. The allergic score was defined as the sum of the four individual scores and ranged from 0 to 8. In some experiments, cromolyn disodium (Sigma-Aldrich; 15 mg/kg in 100 μL saline) was given intraperitoneally 30 min before oral saline or OVA challenge. After the last challenge (tenth), ileum and colon samples were collected.

Milk-induced food allergy was induced in BALB/c mice. WT mice were sensitized orally with 20 mg of skim milk and 2.6 μg of cholera toxin as an adjuvant in a total volume of 100 μl sterile saline and boosted five times at three to four day intervals. After sensitization, mice were challenged orally with 20 mg of skim milk in 100 μl saline or saline only five times at three to four day intervals. Allergic symptoms were assessed by the scoring scheme as OVA-induced food allergy. After the last challenge (fifth), ileum and colon samples were collected.

### Experimental colitis mouse model

WT mice were given 2% DSS (36–50 kDa; MP Biomedicals, Santa Ana, CA) in drinking water *ad libitum* for 7 days and then withdrawn for 3 days. The control group received only water. Disease activity index was monitored daily. Disease activity index was determined as follows: normal stool = 0, soft but formed = 1, very soft = 2, diarrhea = 3, and dysenteric diarrhea = 4. Upon necropsy, tissue samples were collected for histological analysis.

### OVA-induced allergic airway inflammation

WT mice were sensitized with OVA on day 1 and day 14 as described above. The sensitized mice were challenged intranasally with OVA (100 μg in 20 μl saline) or saline every other day from day 28. Mice were euthanized 24 h after the last challenge (tenth). Lungs were inflated at 80 cm H_2_O with saline, excised from the chest, and collected for histological analysis.

### Allergic skin inflammation

WT mice were sensitized by subcutaneous injection into the ear lobe with 1.25 μg of anti-DNP-specific IgE (SPE-7; Sigma-Aldrich). Mice were then challenged 24 h later with 20 μl of 0.2% 2,4-dinitrofluorobenzene (DNFB; Nacalai Tesque, Kyoto, Japan) in acetone:olive oil (4:1) or vehicle alone. Ear tissues were collected 10 days after challenge for histological examination.

### Urine collection

Urine samples from experimental model mice were collected for 24 h in metabolic cages. In some experiments, the nonselective COX inhibitor, indomethacin (Sigma-Aldrich; 5 mg/kg in 100 μL saline); the selective COX-1 inhibitor, SC-560 (Cayman Chemical, Ann Arbor, MI; 10 mg/kg in 100 μL saline); or the selective COX-2 inhibitor, NS-398 (Cayman Chemical; 10 mg/kg in 100 μL saline), was orally administered 2 days before urine sampling.

Urine specimens from human allergic patients and healthy volunteers were obtained at the University of Tokyo Hospital. Urine samples were stored at −80 °C until analysis.

### Histology

Excised tissues were fixed in 4% paraformaldehyde for 24 h and embedded in paraffin. Tissue sections (2 μm) of the intestine, lung, and ear were stained with hematoxylin and eosin (H&E) and chloroacetate esterase (CAE). CAE-positive mast cells were counted in 25 randomly selected fields (×40 magnification) per section. Neutrophils and eosinophils were also counted in 25 randomly selected fields (×40 magnification) per H&E section.

### Immunofluorescence

Immunofluorescence of H-PGDS, c-kit and COX-2 was performed, as described previously^29^. Briefly, excised colons were fixed in 4% paraformaldehyde at 4 °C for 2 h. Cryosections (4 μm) were blocked with PBS containing 0.05% Triton X-100 and 3% normal goat serum albumin at room temperature for 15 min. The sections were then immunostained using primary antibodies against H-PGDS (Cayman Chemicals, 1:500, rabbit), c-kit (Merck Chemical, Darmstadt, Germany, 1:125, rat) and COX-2 (Santa Cruz Biotechnology, Santa Cruz, CA, 1:100, goat) at 4 °C overnight. The secondary antibodies, goat anti-rabbit Alexa 568 and goat anti-rat Alexa 488 or donkey anti-goat Alexa 488 (Invitrogen, Carlsbad, CA, 1:500), were incubated at room temperature for 2 h. Images were captured using an Eclipse E800 fluorescence microscope (Nikon, Tokyo, Japan).

### BMMC preparation

For preparation of BMMCs, bone marrow cells were isolated from the humerus, femur, and tibia of C57BL/6J mice and cultured in RPMI-1640 (Invitrogen) supplemented with 10% FBS (Nichirei Biosciences, Tokyo, Japan), 50 μM 2-ME, 2 mM L-glutamine, 100 U/ml penicillin, 100 mg/ml streptomycin, and 10 ng/ml mouse IL-3 (Prospec, Ness Ziona, Israel) for up to six weeks at 37 °C in 5% CO_2_.

### *In vitro* degranulation assay

After 4 h starvation, BMMCs (1 × 10^6^) were sensitized overnight with 100 ng/ml of anti-DNP mouse IgE (SPE-7; Sigma-Aldrich) and then stimulated with 100 ng/ml of DNP-BSA (EMD Chemicals, Gibbstown, NJ). The stimulated cells were incubated at 37 °C in 5% CO_2_ for 4 h, and the supernatants were used for measurement of PGD_2_.

### Adoptive transfer of BMMCs to Kit^*W-sh/W-sh*^ mice

BMMCs (1–5 × 10^6^) were injected into the tail vein of *Kit* ^*W-sh/W-sh*^ mice. After 14 days, urine was collected for 24 h in metabolic cages for further analysis.

### Comprehensive analysis of lipid metabolites in the urine

Liquid chromatography coupled with tandem mass spectrometry (LC-MS/MS)-based comprehensive analysis of lipid metabolites was performed by using a LCMS-TQ8030 triple-quadruple tandem mass spectrometer (Shimadzu, Kyoto, Japan) and a software method package for lipid mediators (Shimadzu) according to the manufacturer’s instructions. This software is a program package for the simultaneous analysis of 130 lipid products with 13 deuterium internal standards (Supplemental Table 1), and the metabolites were identified and quantified by multiple reactions monitoring (MRM). Data were analyzed by LabSolutions LCMS Ver. 5.65 (Shimadzu).

### Measurement of urinary tetranor-PGDM and tetranor-PGEM

Urinary tetranor-PGDM and tetranor-PGEM were measured by LC-MS/MS, as described previously^30^. Briefly, mouse and human urine (0.4 ml) were both diluted up to 1 ml with water acidified by 1 mol/l HCl (final pH ~ 3), and then 5 ng of d6-tetranor-PGDM and d6-tetranor-PGEM (Cayman Chemical) were added as internal standards. The samples were loaded on a Sep-Pak Vac 3 cc cartridge (Waters, Milford, MA) for solid-phase extraction. The analyte was eluted from the cartridge with 3 ml of ethyl acetate, dried under vacuum, dissolved in 100 μl of 10% acetonitrile in water, and injected for LC-MS/MS analysis. LC separation was carried out using a mobile phase consisting of 0.02% acetic acid (solvent A) and acetonitrile (solvent B). Chromatographic elution was performed using a gradient of 5–30% solvent B for the first 10 min followed by 5 min at 95% solvent B at a flow rate 0.4 ml/min. The LC-MS/MS was operated in the negative ion mode. Transitions monitored were *m/z* 327 → 309 for the endogenous materials (tetranor-PGDM and tetranor-PGEM) and *m/z* 333 → 315 for the internal standards (d6-tetranor-PGDM and d6-tetranor-PGEM). Urinary creatinine concentration was measured using a kit (Wako, Tokyo, Japan), and the urinary tetranor-PGDM and tetranor-PGEM were expressed as ng/mg creatinine.

### PGD_2_ measurement

The culture media of BMMCs (0.4 ml) were diluted up to 1 ml with water acidified by 1 mol/l HCl (final pH ~ 3), and then 5 ng of d4-PGD_2_ (Cayman Chemical) was added as internal standards. The samples were loaded on a Sep-Pak Vac 3 cc cartridge for solid-phase extraction. The analyte was eluted from the cartridge with 5 ml of ethyl acetate, dried under vacuum, dissolved in 100 μl of 10% acetonitrile in water, and injected for LC-MS/MS analysis. LC separation was carried out using a mobile phase consisting of 0.02% acetic acid (solvent A) and acetonitrile (solvent B). Chromatographic elution was performed using a gradient of 5–30% solvent B for the first 10 min, 30–65% solvent B for 8 min, 65–90% solvent B for 5 min, followed by 10 min at 90% solvent B at a flow rate 0.4 ml/min. The LC-MS/MS was operated in the negative ion mode. Transitions monitored were *m/z* 351 → 315 for PGD_2_ and *m/z* 355 → 319 for d4-PGD_2_.

### Statistics

Data are expressed as mean ± SEM, and all experiments were repeated at least three times. Statistical analyses were performed with Prism 6.0 (GraphPad Software, La Jolla, CA). Statistical differences were analyzed by Student’s *t* test for two-group comparisons, one-way ANOVA with Tukey’s test or Dunnett’s test for multiple-group comparisons, and by the Kruskal–Wallis test with the Steel–Dwass test for the allergic scoring and human urinary metabolites. The relationships between urinary metabolites and allergic score or colon mast cells were evaluated by the Spearman’s rank correlation coefficient. Statistical significance was defined as *P* < 0.05.

### Data availability

All data generated or analysed during this study are included in this published article and its Supplementary Information files.

## Electronic supplementary material

Supplemental information
