# Supplementary material for: Prostaglandin D2 metabolite in urine is an index of food allergy
Source: Sci Rep. 2017 Dec 15;7:17687. doi: 10.1038/s41598-017-17798-w (PMC5732293; doi:10.1038/s41598-017-17798-w)
Supplement: Supplementary file 1 — Supplemental information [file 41598_2017_17798_MOESM1_ESM.pdf]

Prostaglandin D_2 metabolite in urine is an index of food allergy

Shingo Maeda, Tatsuro Nakamura, Hiroaki Harada, Yuri Tachibana, Kosuke Aritake, Tatsuo Shimosawa, Yutaka Yatomi, and Takahisa Murata

Online data supplement

Supplementary Figures

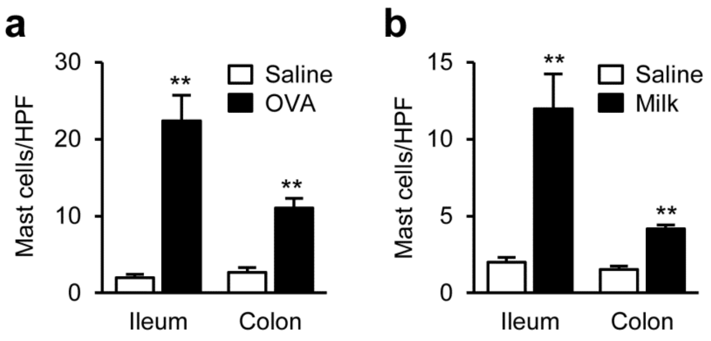

Supplemental Figure 1. Intestinal mast cell number in murine models of food allergy. (**a** and **b**) Mean number of CAE-positive mast cells per high-power field (HPF) in the ileum and colon after challenges of OVA (tenth, **a**) and milk (fifth, **b**), respectively (n = 4–5). Student's t test was used. $^{**}P < 0.01$ vs. saline-challenged mice.

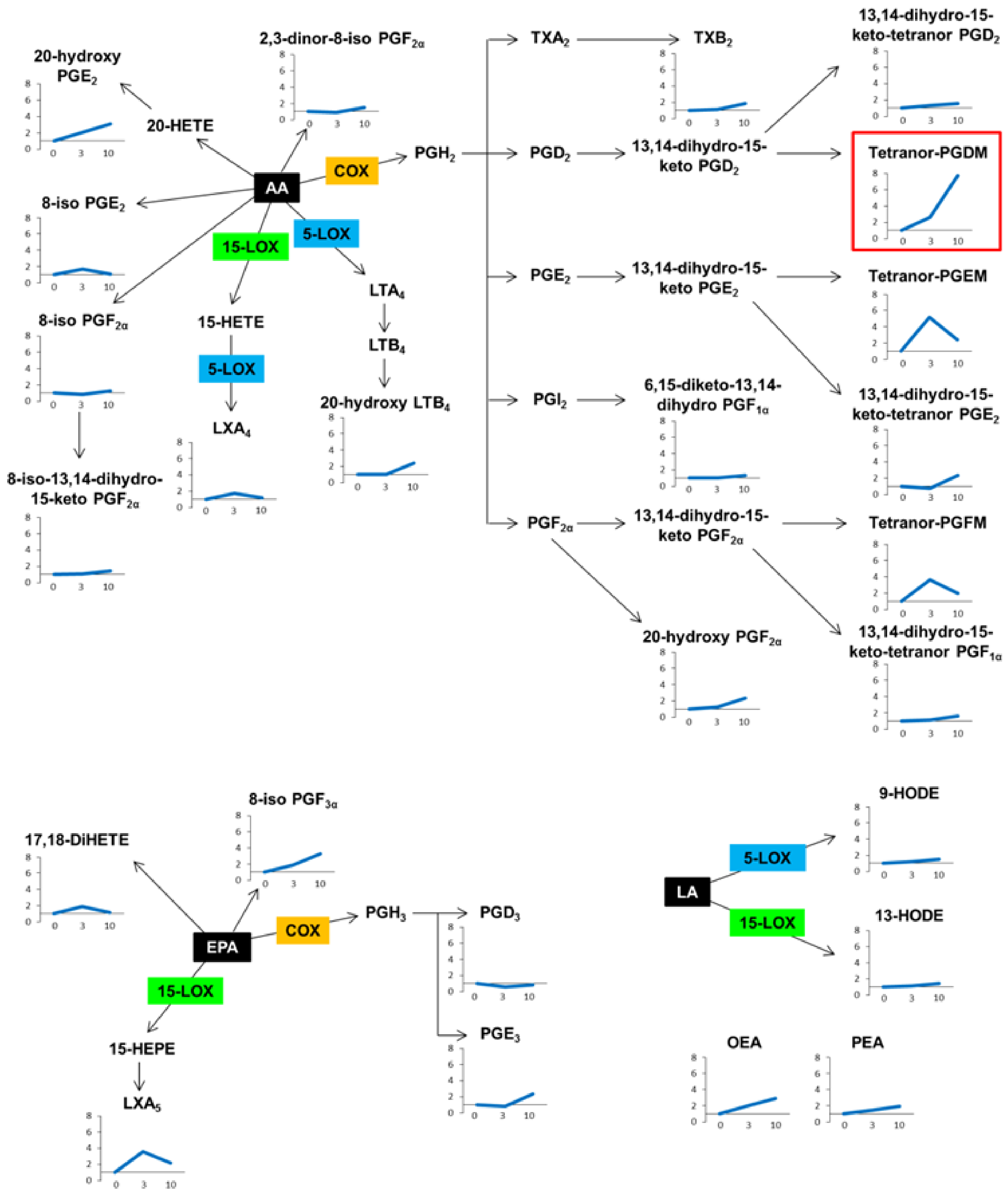

Supplemental Figure 2. Urinary lipid metabolites in mice with oral OVA-induced food allergy. Lipid metabolites derived from arachidonic acid (AA), eicosapentaenoic acid (EPA), linoleic acid (LA), and other lipids detected in mouse urine. Urine samples were obtained from unchallenged mice and third or tenth OVA-challenged mice (*n* = 7 each). The horizontal axis shows the number of OVA challenges. The vertical axis shows the ratio to the urine of unchallenged mice.

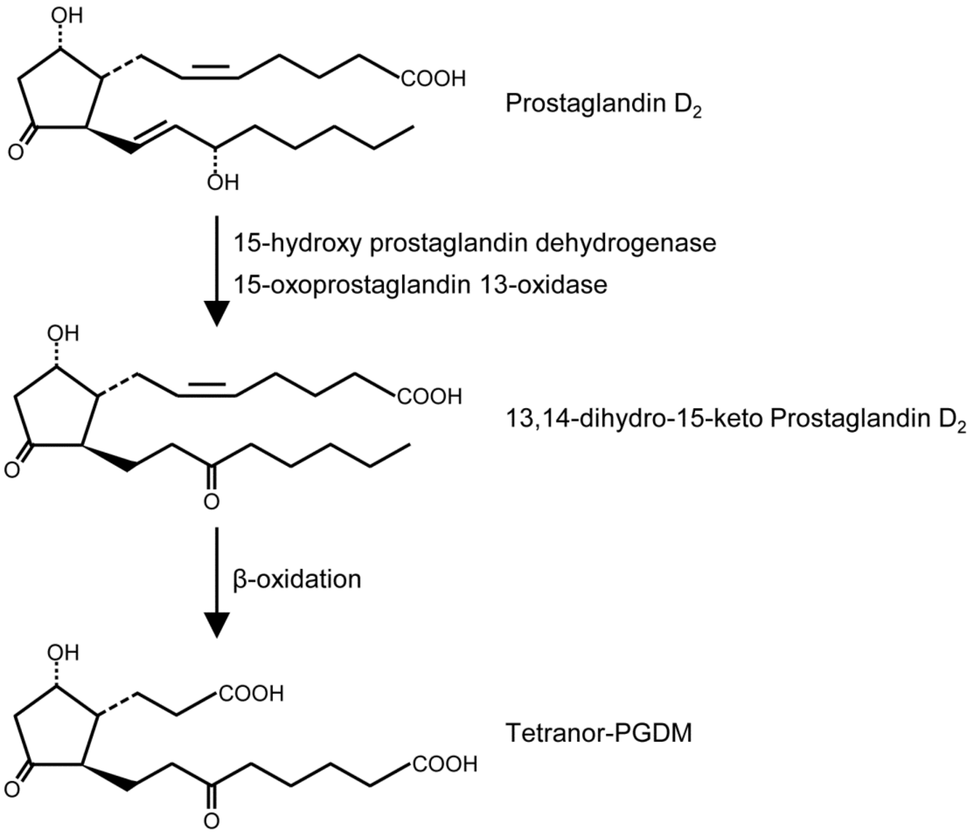

Supplemental Figure 3. Metabolic pathway of tetranor-PGDM. Prostaglandin D_2 (PGD_2) can be metabolized to the 13,14-dihydro-15-keto prostaglandin D_2 by the enzymes 15-hydroxy prostaglandin dehydrogenase and 15-oxoprostaglandin 13-oxidase. The 13,14-dihydro-15-keto prostaglandin D_2 can be transformed to the urinary metabolite tetranor-PGDM via β-oxidation.

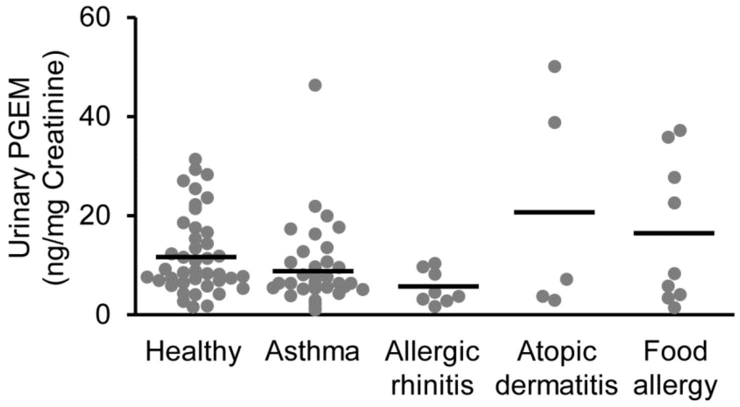

Supplemental Figure 4. Tetranor-PGEM levels in human urine. Urinary tetranor-PGEM in healthy human volunteers (*n* = 39) and patients with asthma (*n* = 37), allergic rhinitis (*n* = 8), atopic dermatitis (*n* = 5), and food allergy (*n* = 9). The mean of the levels of tetranor-PGEM in each group is denoted by horizontal lines. Mean values were compared by the Kruskal−Wallis test. There was no significant difference among groups.

Supplemental Table 1. Lipid mediators and metabolites in a method package (Shimadzu).

No.	Type	Product
1	LA	13(S)-HODE
2	LA	9(S)-HODE
3	LA	13-OxoODE
4	LA	9-OxoODE
5	ALA	9(S)-HOTrE
6	ALA	13(S)-HOTrE
7	EDA	(±)15-HEDE
8	EDA	15-OxoEDE
9	AA	tetranor-PGFM
10	AA	tetranor-PGEM
11	AA	tetranor-PGDM
12	AA	20-hydroxy Prostaglandin $F_{2\alpha}$
13	AA	20-hydroxy Prostaglandin E_2
14	AA	18-carboxy dinor LTB_4
15	AA	13,14-dihydro-15-keto-tetranor Prostaglandin $F_{1\beta}$
16	AA	2,3-dinor-8-iso Prostaglandin $F_{2\alpha}$
17	AA	13,14-dihydro-15-keto-tetranor Prostaglandin $F_{1\alpha}$
18	AA	6-keto-Prostaglandin $F_{1\alpha}$
19	AA	13,14-dihydro-15-keto-tetranor Prostaglandin D_2
20	AA	20-carboxy LTB_4

21	AA	20-hydroxy LTB_4
22	AA	13,14-dihydro-15-keto-tetranor Prostaglandin E_2
23	AA	6,15-diketo-13,14-dihydro Prostaglandin $F_{1\alpha}$
24	AA	$iPF_{2\alpha}$-IV
25	AA	8-iso-15(R)-Prostaglandin $F_{2\alpha}$
26	AA	8-iso Prostaglandin $F_{2\alpha}$
27	AA	Thromboxane B_2
28	AA	11β-Prostaglandin $F_{2\alpha}$
29	AA	5-$iPF_{2\alpha}$-VI
30	AA	8-iso-15-keto Prostaglandin $F_{2\alpha}$
31	AA	Prostaglandin $F_{2\alpha}$
32	AA	8-iso-13,14-dihydro-15-keto Prostaglandin $F_{2\alpha}$
33	AA	8-iso Prostaglandin E_2
34	AA	Prostaglandin E_2
35	AA	11-dehydro Thromboxane B_2
36	AA	15-keto Prostaglandin $F_{2\alpha}$
37	AA	Prostaglandin D_2
38	AA	11β-13,14-dihydro-15-keto Prostaglandin $F_{2\alpha}$
39	AA	15-keto Prostaglandin E_2
40	AA	14,15-LTC_4, Eoxin C_4, EXC_4
41	AA	13,14-dihydro-15-keto Prostaglandin $F_{2\alpha}$, PGFM
42	AA	5(S),6(R)-Lipoxin A_4

43	AA	13,14-dihydro-15-keto Prostaglandin E_2
44	AA	Leukotriene D_4
45	AA	14,15-LTE_4, Eoxin E_4
46	AA	13,14-dihydro-15-keto Prostaglandin D_2
47	AA	Leukotriene C_4
48	AA	Leukotriene E_4
49	AA	Leukotriene F_4
50	AA	8-iso Prostaglandin A_2
51	AA	Prostaglandin A_2
52	AA	11-trans LTC_4
53	AA	11-trans LTE_4
54	AA	Prostaglandin J_2
55	AA	Prostaglandin B_2
56	AA	8,12-iso-i$PF_{2\alpha}$-VI 1,5- lactone
57	AA	8(S),15(S)-DiHETE
58	AA	6-trans LTB_4
59	AA	5(S),15(S)-DiHETE
60	AA	Leukotriene B_4
61	AA	13,14-dihydro-15-keto Prostaglandin J_2
62	AA	12-oxo LTB_4
63	AA	N-acetyl LTE_4
64	AA	(±)14,15-DHET

65	AA	12(S)-HHTrE
66	AA	(±)11,12-DHET
67	AA	(±)8,9-DHET
68	AA	20-carboxy arachidonic acid
69	AA	(±)5,6-DHET
70	AA	15-deoxy-delta12,14-PGJ_2
71	AA	20-HETE
72	AA	(±)18-HETE
73	AA	(±)17-HETE
74	AA	(±)16-HETE
75	AA	15(S)-HETE
76	AA	11(S)-HETE
77	AA	8(S)-HETE
78	AA	12(S)-HETE
79	AA	5(S)-HETE
80	AA	12-OxoETE
81	AA	(±)5,6-DHET-lactone
82	AA	(±)14(15)-EET
83	AA	5-OxoETE
84	AA	(±)11(12)-EET
85	AA	(±)8(9)-EET
86	AA	(±)5(6)-EET

87	AA	Arachidonic Acid (AA)
88	DGLA	8-iso Prostaglandin $F_{1\alpha}$
89	DGLA	8-iso Prostaglandin $F_{1\beta}$
90	DGLA	8-iso Prostaglandin E_1
91	DGLA	Prostaglandin E_1
92	DGLA	Prostaglandin D_1
93	DGLA	8-iso Prostaglandin A_1
94	DGLA	15(S)-HETrE
95	EPA	8-iso Prostaglandin $F_{3\alpha}$
96	EPA	Prostaglandin E_3
97	EPA	Prostaglandin D_3
98	EPA	Lipoxin A_5
99	EPA	(±)17,18-DiHETE
100	EPA	(±)14,15-DiHETE
101	EPA	5,6-DiHETE
102	EPA	18(S)-HEPE
103	EPA	15(S)-HEPE
104	EPA	12(S)-HEPE
105	EPA	5(S)-HEPE
106	EPA	Eicosapentaenoic Acid(EPA)
107	DHA	Resolvin D_1
108	DHA	(±)16-HDoHE

109	DHA	(±)17-HDoHE
110	DHA	(±)13-HDoHE
111	DHA	(±)10-HDoHE
112	DHA	(±)14-HDoHE
113	DHA	(±)11-HDoHE
114	DHA	(±)7-HDoHE
115	DHA	(±)8-HDoHE
116	DHA	(±)4-HDoHE
117	DHA	Docosahexaenoic Acid (DHA)
118	EA	$PGF_{2\alpha}$ Ethanolamide
119	EA	PGE_2 Ethanolamide
120	EA	PGE_1 ethanolamide
121	EA	PGD_2 Ethanolamide
122	EA	LTB4 ethanolamide
123	EA	(±)14(15)-EET ethanolamide
124	EA	(±)11(12)-EET ethanolamide
125	EA	(±)8(9)-EET ethanolamide
126	EA	(±)5(6)-EET ethanolamide
127	EA	PEA (palmitoyl ethanolamide)
128	EA	Arachidonoyl ethanolamide
129	EA	OEA (oleoyl ethanolamide)
130		PAF C-16

131	ISTD	tetranor-PGEM-d_6
132	ISTD	6-keto-Prostaglandin $F_{1\alpha}$-d_4
133	ISTD	Thromboxane B_2-d_4
134	ISTD	Prostaglandin $F_{2\alpha}$-d_4
135	ISTD	Prostaglandin E_2-d_4
136	ISTD	Prostaglandin D_2-d_4
137	ISTD	Leukotriene C_4-d_5
138	ISTD	Leukotriene B_4-d_4
139	ISTD	5(S) HETE-d_8
140	ISTD	12(S) HETE-d_8
141	ISTD	15(S) HETE-d_8
142	ISTD	PAF C-16-d_4
143	ISTD	Oleoyl Ethanolamide-d_4

LA, linoleic acid. ALA, α-linolenic acid. EDA, eicosadienoic acid. DGLA, dihomo-γ-linolenic acid. AA, arachidonic acid. EPA, eicosapentaenoic acid. DHA, docosahexaenoic acid. EA, ethanolamide. ISTD, internal standard.
